# High Prevalence of Antibiotic Resistance among Opportunistic Pathogens Isolated from Patients with COVID-19 under Mechanical Ventilation: Results of a Single-Center Study

**DOI:** 10.3390/antibiotics10091080

**Published:** 2021-09-06

**Authors:** Chiara Temperoni, Luca Caiazzo, Francesco Barchiesi

**Affiliations:** 1 Malattie Infettive, Azienda Ospedaliera Ospedali Riuniti Marche Nord, 61121 Pesaro, Italy; luca.caiazzo@ospedalimarchenord.it (L.C.); f.barchiesi@univpm.it (F.B.); 2 Dipartimento di Scienze Biomediche e Sanità Pubblica, Università Politecnica delle Marche, 60121 Ancona, Italy

**Keywords:** antibiotic resistance, epidemiology, nosocomial infections, COVID-19, mechanical ventilation, new antibiotics

## Abstract

The effect of the COVID-19 pandemic on antibiotic resistance diffusion in healthcare settings has not been fully investigated. In this study we evaluated the prevalence of antibiotic resistance among opportunistic pathogens isolated from patients with COVID-19 under mechanical ventilation. An observational, retrospective, analysis was performed on confirmed cases of COVID-19 patients who were admitted to the ICU department of San Salvatore Hospital in Pesaro, Italy, from 1 February 2021 to 31 May 2021. We considered all consecutive patients aged ≥ 18, under mechanical ventilation for longer than 24 h. Eighty-nine patients, 66 (74.1%) men and 23 (25.9%) women, with a median age of 67.1 years, were recruited. Sixty-eight patients (76.4%) had at least one infection, and 11 patients (12.3%) were colonized, while in the remaining 10 patients (11.2%) neither colonization nor infection occurred. In total, 173 microorganisms were isolated. There were 73 isolates (42.2%) causing bacterial or fungal infections while the remaining 100 isolates (57.8%) were colonizers. Among Gram-negative bacteria, *E. coli*, *A. baumannii* and *K. pneumoniae* were the most common species. Among Gram-positive bacteria, *S. aureus* and *E. faecalis* were the most common species. Overall, there were 58/105 (55.2%) and 22/59 (37.2%) MDR isolates among Gram-negative and Gram-positive bacteria, respectively. The prevalence of an MDR microorganism was significantly higher in those patients who had been exposed to empiric antibiotic treatment before ICU admission. In conclusion, we found a high prevalence of antibiotic resistance among opportunistic pathogens isolated from patients with COVID-19 under mechanical ventilation.

## 1. Introduction

The emergence of Coronavirus 2 (SARS Cov2) infection had a big impact on healthcare organization and on antibiotics consumption, determining an elevated risk of increasing antimicrobial resistance development, especially in those patients requiring Intensive Care Unit (ICU) admission [1,2]. Patients under mechanical ventilation are at high risk of aerosol and droplet transmission; moreover, all the supplies used to prevent SARS CoV2 contamination increase the transmission of pathogens, thereby increasing the risk of colonization and infection due to multi-drug-resistant (MDR) microorganisms [3,4,5,6]. This fact suggests the need for a specific antimicrobial stewardship program to be implemented in this setting.

In this study, we aimed to describe the prevalence of antibiotic resistance among opportunistic pathogens isolated from COVID-19 patients under mechanical ventilation prospectively seen in ICU of Pesaro Hospital.

## 2. Results

**Figure 1:**
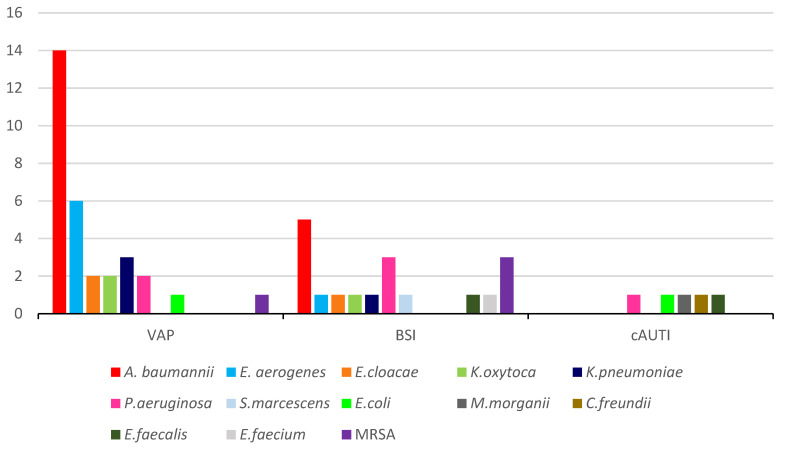
MDR pathogens (n°) causing VAP, BSI and cAUTI.

In this 4-month observational study, we recruited 89 patients; 66 (74.1%) men and 23 (25.9%) women. Median age was 67.1 years (IQR 42-83). The most frequent comorbidities were hypertension (52.8%), obesity (39.3%), diabetes (19.1%) and chronic kidney disease (11.3%). The median length of hospital stay (i.e.,: days from hospital admission to discharge or death), length of ICU stay (i.e.,: days from ICU admission to discharge or death), and length of mechanical ventilation were 23 days (IQR 13–30), 21 days (IQR 11–30), and 19 days (IQR 10–27), respectively. A total of 68 patients (76.4%) had at least one infection: 46 patients (51.6%) had one infection, 22 patients (24.7%) had ≥2 infections. There were 48 ventilator-associated pneumonia (VAP) (52.1%), 29 bloodstream infections (BSI) (31.5%) and 15 catheter-associated urinary tract infections (cAUTI) (16.3%) (Table 1). The majority of VAP and BSI were caused by MDR microorganisms, while the majority of cAUTI were caused by susceptible isolates (Table 1 and Figure 1).

A total of 11 patients (12.3%) were colonized, while in the remaining 10 patients (11.2%) neither colonization nor infection occurred in the study period (i.e.,: no microbiological isolation or clinically manifest infection occurred during hospitalization).

**Figure 2:**
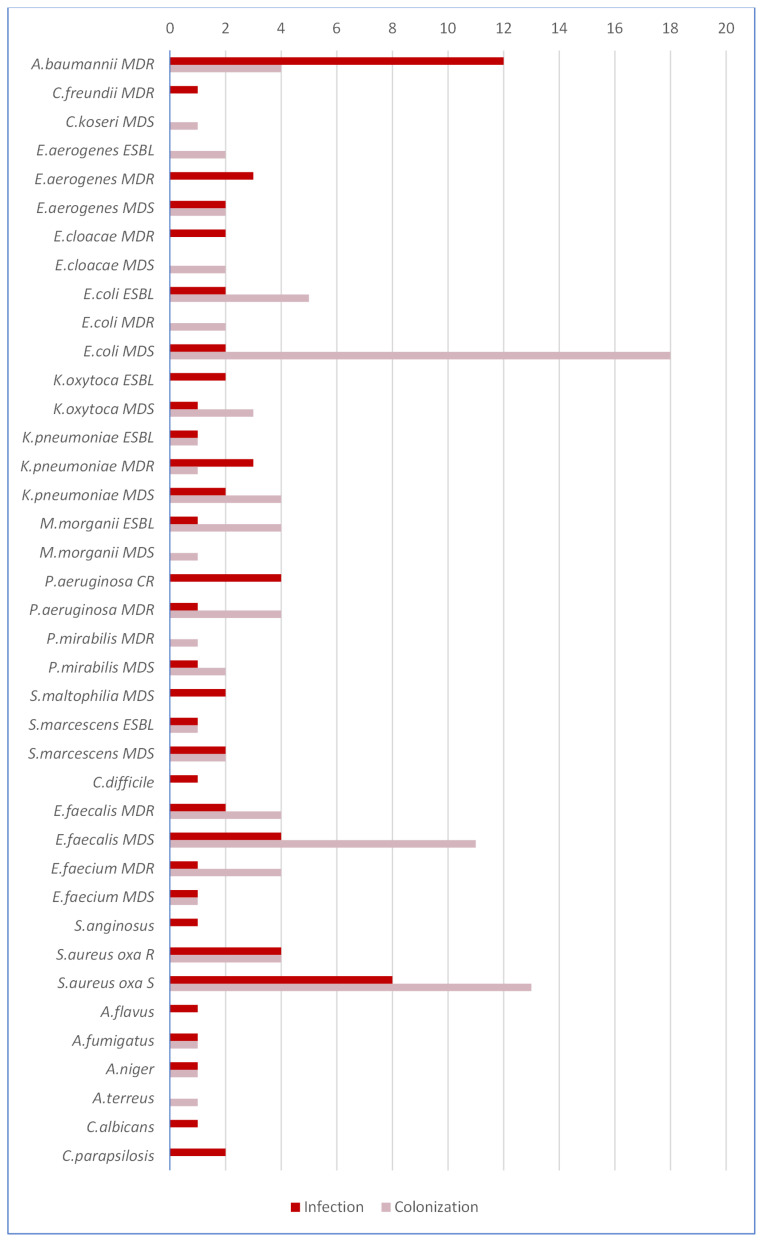
Isolates recovered from COVID-19 patients. S, Susceptible; MDR, Multi-Drug-Resistant; R, Resistant; MDS, Multi-Drug-Susceptible; ESBL, Extended Spectrum-Beta-Lactamase; CR, Carbapenem-Resistant.

In total, 173 microorganisms were isolated. There were 73 isolates (42.2%) causing bacterial or fungal infections while the remaining 100 isolates (57.8%) were colonizers (Figure 2).

There were 105 Gram-negative bacteria (60.7%), 59 Gram-positive bacteria (34.1%) and nine fungi (5.2%). Among Gram-negative bacteria, *E. coli*, *A. baumannii* and *K. pneumoniae* were the most common species (29.5%, 15.2% and 11.4%, respectively). Among Gram-positive bacteria, *S. aureus* and *E. faecalis* were the most common species (35.5% and 18.6%, respectively). Six strains belonging to the genus *Aspergillus* (two *A. fumigatus*, two *A. niger* one *A. flavus* and one *A. terreus*) were isolated among filamentous fungi. Three isolates of *Candida* spp. (two *C. parapsilosis* and one *C. albicans*) were recovered from blood. Overall, there were 58/105 (55.2%) and 22/59 (37.2%) MDR isolates among Gram-negative and Gram-positive bacteria, respectively. The median isolation latency from ICU admission was 9.7 days (IQR 5–13) for MDS microorganisms vs. 14.9 days (IQR 7–21) for MDR microorganisms (*p* < 0.001). A total of 51 out of 89 patients (57.3%) had been exposed to at least three days of empiric antibiotic treatment before ICU admission. The prevalence of an MDR organism as first isolation during ICU stay was significantly higher in antibiotic-exposed (12/51, 23.5%) than -unexposed patients (2/38, 5.2%) (*p* < 0.05).

## 3. Discussion

The effect of the COVID-19 pandemic on antibiotic resistance diffusion in healthcare settings has not been fully investigated. Healthcare facilities used to prevent SARS CoV2 spread have increased the risk of transmission of MDR [7,8,9,10].

In this study, we evaluated the prevalence of antibiotic resistance among opportunistic pathogens isolated from patients with COVID-19 under mechanical ventilation. We found that a large amount of our patients harbored MDR pathogens, especially those who had been exposed to antibiotics in the days before ICU admission.

We found a greater proportion of MDR strains among Gram-negative than among Gram-positive bacteria. We observed a high prevalence of *A. baumannii* carbapenem-resistant (CRAB) colonization with an infection rate of 75% (12/16). In a previous report conducted in 34 patients with SARS CoV2 who acquired CRAB during hospital stay, the infection rate was 59% [11]. As expected, 100% of isolates of *P. aeruginosa* were MDR, including four carbapenem-resistant (CR) strains. Similarly, 50% of *K. pneumoniae* isolates were MDR, while only 31% of isolates of *E. coli* resulted MDR. Isolation of MDR Gram-negative bacteria is frequently related to protracted use of external devices such as urinary catheters, central venous lines, to prolonged length of stay and to mechanical ventilation [12]. Drug-resistant bacteria present a serious threat to human health; the increasing antibiotic resistance especially with the emergence of CRE is a big deal in clinical practice. Carbapenem-resistant *K. pneumoniae* and *P. aeruginosa* have risen by 26.8% and 15.8%, respectively, in Italy [13]. So far, there have been few treatment options for dealing with infections caused by these difficult opportunistic pathogens. Lately, several molecules have become available including ceftazidime-avibactam, meropenem-varbobactam and cefiderocol [14,15,16]. These new molecules have been often employed in our series of patients: in particular, ceftazidime-avibactam and cefiderocol (the latter in compassionate use) were often used for treating VAP and BSI.

Among Gram-positive bacteria, *S. aureus* and *E. faecalis* were the most common species: MRSA accounted for 27.5% (8/29) while all isolates of *E. faecalis* were multi-drug-susceptible. As expected, *E. faecium*, the third most common species among Gram-positive bacteria, showed to be MDR in 71.4% (5/7) of the cases.

A remarkable interest must be focused on drug resistance among Gram-positive bacteria, considering that MRSA and glycopeptide-resistant enterococci are increasing and taking into account that in the last 10 years the pipeline for Gram-positive bacteria has been poor [17,18,19].

In 2012, ceftaroline fosamil was approved by EMA. It is a parental cephalosporin with expanded Gram-positive activity, including vancomycin-resistant *S. aureus* (VRSA) and MRSA [19].

ICU COVID-19 patients are usually intubated and often receive multiple antibiotic courses, thus increasing the risk of MDR microorganism isolation. It has been reported that up to 70% of hospitalized patients with COVID-19 received antibiotics, but less than 10% had bacterial infection, thus suggesting a poor stewardship [20]. It has also been shown that bacterial coinfection is rarer than in influenza [21]. Moreover, one study found that administration of antibiotics before ICU transfer, had little effects [22]. Similarly, we found that pre-exposure to a short course of antibiotic therapy facilitates the isolation of MDR microorganisms in these patients.

This study has several limitations. First, since our data come from a single-center experience, our findings may not be relevant to other patient populations. However, our study group was quite homogeneous, being all patients under mechanical ventilation. Second, since the number of patients, and consequently the number of isolates, are quite low, the statistical power of the study can be certainly weakened. Despite this, during the study (four months) the patients consecutively admitted in ICU were clinically examined from the ID consultant every other day, thus making clinical and microbiological evaluation rather effective. Third, the lack of a control group precludes any causality inference in this setting. It must be noted, however, that during the study period our center was completely converted to a COVID hospital, including the ICU department, while COVID-19 negative patients were transferred to other hospitals, thus making any comparison difficult. Finally, since the main purpose of our work was to describe the microbial population in these patients, clinical aspects were not included in the present analysis.

## 4. Materials and Methods

### 4.1. Patients and Data Collections

An observational, retrospective analysis was performed on confirmed cases of COVID-19 patients who were admitted to the ICU department of S. Salvatore Hospital in Pesaro from 1 February 2021 to 31 May 2021. We considered all consecutive patients aged ≥18, under mechanical ventilation for longer than 24 h. For all patients, data concerning demographics (age, gender), epidemiology, comorbidities, microbiologic results (blood, urine and respiratory samples), length of hospital stay, ICU stay and length of mechanical ventilation were collected. The Ethics Committee of Marche Region does not require formal approval for observational retrospective studies that do not involve the use of drugs. The work was carried out in accordance with The Code of Ethics of the World Medical Association for experiments involving humans (Declaration of Helsinki) and research on health databases (Declaration of Taipei). Patients and caregivers gave their written consent to use their personal data at the admission into the Hospital. Anonymity of patients was guaranteed during the whole process of data analysis and results reporting.

### 4.2. Definitions

Ventilator-associated pneumonia (VAP) refers to pneumonia that arises more than 48 h after endotracheal intubation [23]. Bloodstream infection (BSI) was defined as the occurrence of at least one positive blood culture positive for bacteria or fungi, in a patient with temporally related clinical signs and symptoms of infection, performed >48 h after ICU admission. One episode of coagulase-negative staphylococci (CoNS) bacteremia was defined as the presence of CoNS in two or more blood cultures, while its presence in one single blood culture was considered as a contaminant [24]. Catheter-associated urinary tract infections (cAUTI) were defined as per CDC [25] criteria as an infection that occurred while the patient had urinary catheter in place within 2 days of catheterization associated with fever > 38 °C and positive urinary culture of more >10^5^ CFUs. Microbiologic results were independently reviewed by two infectious disease specialists who categorized the isolated pathogens as colonizers when clinical features, as described above, were not occurring. Bacteria were considered MDR when resistance to three or more antimicrobial classes occurred [26].

### 4.3. Statistical Analysis

Continuous variables were expressed as median (IQR) and compared with the Mann–Whitney U test when data were normally distributed; categorical variables were expressed as number (%) and compared by χ^2^ test. A two-sided α of less than 0.05 was considered statistically significant. All the statistical analyses were supported by SPSS (Statistical Package for the Social Sciences) version 25.0 software (SPSS Inc., Chigago, IL, USA).

## 5. Conclusions

In conclusion, critically ill patients with COVID-19 are often colonized/infected with difficult-to-treat opportunistic pathogens. Gram-negative bacteria predominate over Gram-positive. We found that a large amount of our patients harbored MDR pathogens especially those who had been exposed to antibiotics in the days before ICU admission. Clinicians must be aware about this insidious threat and the therapeutical opportunities offered by new molecules.

## Figures and Tables

**Table 1:** Infections diagnosed during the study period.

Types of Infections	N° of Infections	N° of Infections Caused by MDR Pathogens	%
VAP	48	31	64.5
BSI	29	18	62
cAUTI	15	5	33.3

## Data Availability

The data would be available upon request.
